# Protective Effect of Shengmaiyin in Myocardial Hypertrophy-Induced Rats: A Genomic Analysis by 16S rDNA

**DOI:** 10.1155/2022/3188292

**Published:** 2022-09-07

**Authors:** Sitong Ming, Mo Kan, Liu Liu, Zhuang Zhang, Xiaoran Liu, Yaxin Liu, Zhen Li, Yanhong Zhang, Qihang Pang, Jianan Lin, Hui Li, Qing Yang, Xin Sui, Xiaobo Qu, Na Li

**Affiliations:** ^1^Jilin Provincial Key Laboratory of Biomacromolecules of Chinese Medicine, Jilin Ginseng Academy, Changchun University of Chinese Medicine, Changchun, Jilin 130021, China; ^2^Jilin Province Drug Evaluation Center, Changchun, Jilin 130062, China; ^3^Qian Wei Hospital of Jilin Province, Changchun, Jilin 130012, China

## Abstract

**Background:**

The gut-cardiac axis theory provides new insights into the complex mechanisms of cardiac hypertrophy and provides new therapeutic targets. Cardiac hypertrophy is a risk factor for heart failure. Shengmaiyin (SMY) is a traditional Chinese medicine formula with clear effects in the treatment and prevention of cardiac hypertrophy, but the mechanism by which it improves cardiac hypertrophy is still unclear. Therefore, this study aimed to investigate the protective effect and mechanism of SMY on isoproterenol (ISO)-induced myocardial hypertrophy in rats.

**Methods:**

First, various pharmacodynamic methods were used to evaluate the therapeutic effect of SMY on ISO-induced myocardial hypertrophy in rats. Then, 16S rDNA amplicon sequencing technology was used to study the effect of SMY on the intestinal flora of rats with myocardial hypertrophy. Finally, the mechanism underlying the effect of SMY on cardiac hypertrophy was predicted by bioinformatics network analysis and verified by Western blotting.

**Results:**

SMY increased ejection fraction (EF%) and left ventricular fractional shortening (FS%), ameliorated myocardial cell injury and fibrosis, regulated blood lipids and energy metabolism, and decreased cardiac hypertrophy marker gene expression. The gut microbiota of ISO-induced myocardial hypertrophy rats were significantly changed, while SMY effectively ameliorated the dysbiosis of the intestinal flora in rats with myocardial hypertrophy, especially *Prevotella 9*, *Lactobacillus,* and *Clostridium*. Mechanistic studies have shown that the anticardiac hypertrophy effect of SMY is related to the inhibition of the expression of HIF1*α*/PPAR signalling pathway-related proteins.

**Conclusion:**

SMY significantly improves cardiac function, relieves myocardial cell fibrosis and necrosis, resists cardiac hypertrophy, improves blood lipid metabolism and energy metabolism, regulates intestinal microbial disturbance, and protects the heart.

## 1. Introduction

Myocardial hypertrophy is the compensatory response of the heart to various stimuli. It is an adaptive response of cardiomyocytes under long-term pressure load to maintain the contractile function of the heart when the heart's afterload continues to increase. Basic cell morphology is altered and pathological changes occur. Cardiomyocyte hypertrophy is mainly divided into physiological and pathological hypertrophy [1]. Pathological myocardial hypertrophy is an abnormal change in the structure of the heart caused by various factors, such as hypertension and arrhythmia. If no timely and effective intervention is administered, it eventually progresses to irreversible heart failure [2]. Pathological hypertrophy often manifests as an increased volume of myocardial cells, increased heart mass, myocardial disorganization, and myocardial fibrosis [3]. Furthermore, pathological myocardial hypertrophy is related to ventricular remodelling, which is associated with hypertension, atrial fibrillation, ventricular tachycardia, ventricular fibrillation, and sudden cardiac death [4]. Clinically, angiotensin-converting enzyme inhibitors, angiotensin receptor antagonists, *β*-receptor blockers, and calcium channel blockers are mainly used for the treatment of myocardial hypertrophy [5–7]. However, while these treatments can delay the occurrence and development of myocardial hypertrophy and fibrosis to a certain extent, they cannot significantly delay the development of heart failure [8]. Therefore, it is of great significance and value to identify drugs that inhibit myocardial cell hypertrophy and myocardial fibrosis to prevent and treat myocardial hypertrophy.

Shengmaiyin (SMY) was first reported in Medical Origination by Zhang Yuan, a physician in the Jin Dynasty. It is composed of three traditional Chinese medicines, *Codonopsis pilosula*, *Ophiopogon japonicus*, and *Schisandra chinensis*, and it replenishes qi and nourishes yin. It has a wide range of applications in the treatment of cardiovascular, central nervous system, and endocrine system diseases. Modern pharmacological studies have shown that SMY protects the myocardium and improves heart function [9]. SMY is a commonly used prescription for clinical multisystem diseases, and pharmacological research on its component medicines is ongoing. Modern pharmacological research has shown the following: (1) the main components of *Codonopsis pilosula* are saponins, volatile oils, polysaccharides, and various trace elements. It has a beneficial effect against cardiac diseases such as myocardial ischaemia, ventricular conduction impairment, and reduced cardiac function [10]. (2) The ingredients of *Ophiopogon japonicus* include saponins and isoflavones. Modern pharmacological studies have shown that it has the ability to increase the number of peripheral white blood cells, enhance immune function, protect against hypoxia in cerebral ischaemia, exert antiarrhythmic effects, improve myocardial contractility, improve left ventricular function, and exert antishock, hypoglycaemic, anti-inflammatory, and multiple other pharmacological effects. (3) The main components of *Schisandra* are volatile oils [11].

The gut microbiota is a large community that exists in both humans and rodents [12]. The intestinal microbiota mainly includes *Firmicutes, Bacteroides, Proteus*, and *Verrucobacteria* species, the abundance of which is relatively stable in healthy individuals [13]; however, changes in the abundance of metabolites of the intestinal microbiota are seen in specific groups. For example, changes in the ratios of gut microbiota components are closely related to the occurrence and development of many diseases [14]. Disturbance of the intestinal microbiota can disrupt normal metabolism in the body, enhance the oxidative stress response, and exacerbate systemic inflammation through the translocation of endotoxins to systemic circulation. It is one of the important factors contributing to cardiovascular disease [15]. Studies have reported that hypoperfusion and blood stasis occur in the intestine when the myocardium is hypertrophic, leading to changes in the growth and composition of the intestinal microbiota and the transfer of intestinal microbes and endotoxins to body fluids, leading to systemic inflammation [16]. Thus, the intestinal microbiota and its metabolites have a two-way influence on the occurrence and development of cardiovascular diseases, and the intestinal microbiota may be a new focus of cardiovascular disease research.

## 2. Materials and Methods

### 2.1. Materials

SMY was purchased from Tongrentang (China, Cat no. Z11020372), and isoproterenol hydrochloride (ISO) was obtained from Beijing Solarbio Technology Co., Ltd. (China, Cat no. I8480). Fufangdanshen tablets were purchased from CSPC Holdings Group Co., Ltd. (China, Cat no. Z22022898). A cyclic adenosine monophosphate (cAMP) ELISA kit was obtained from Changchun Baijing Technology Co., Ltd. (China, Cat no. 07/2020), and a free fatty acid (FFA) ELISA kit was purchased from Sino Best Biological Technology Co., Ltd. (China, Cat no. 202104). *β*-actin (Cat no. bs-0061R), adenylate cyclase (ADCY1) (Cat no. bs-3681R), protein kinase A (PKA; Cat no. bs-0520R), glucose transporter (Glut4) (Cat no. bs-0384R), carnitine palmitoyltransferase 1A (CPT1A; Cat no. bs-23779R), PPAR*α* (Cat no. bs-3332R), and hypoxia-inducible factor 1*α* (HIF1*α*; Cat no. bs-0737R) primary antibodies were purchased from Bioss (China). Glut1 (Cat no. 21829-1-AP) and PPAR*γ* (Cat no. 16643-1-AP) primary antibodies were obtained from Proteintech (China). Triglyceride (TG; Cat no. A110-1-1), total cholesterol (T-CHO; Cat no. A111-1-1), low-density lipoprotein-cholesterol (LDL-C; Cat no. A113-1-1), high-density lipoprotein-cholesterol (HDL-C; Cat no. A112-1-1), and glucose (Cat no. A154-1-1) kits were obtained from Nanjing Jiancheng Bioengineering Institute (China). ATP (Cat no. S0026) was purchased from Shanghai Biyuntian Biotechnology Co., Ltd. (China).

### 2.2. Animals

SD rats (8 weeks old) weighing 200 to 210 g (half male and half female) were purchased from Changchun YISI Laboratory Animal Co., Ltd. (China, animal licence number: 201900030925). Animals were housed in a sterile environment at room temperature (22 ± 2°C) and provided access to sufficient water and food. All animal experiments were approved by the Animal Ethics Committee of Changchun University of Traditional Chinese Medicine. We chose SD rats because their growth and development are faster than those of Wistar rats and their adaptability and disease resistance are stronger than those of Wistar rats. The dietary formula for the rats was 73.5% corn, 20% wheat bran, 5% fish meal, 1% grain meal, and 0.5% salt.

### 2.3. Methods

#### 2.3.1. Rat Grouping and Modelling

The rats were randomly divided into the following 5 groups (*n* = 10 each): the blank group (received 0.9% saline), model group (injected with ISO at a decreasing dose (20 mg/kg, 10 mg/kg and 5 mg/kg) for 3 d followed by 3 mg/kg ISO for 4 d), positive group (received 2.90 g/kg Fufangdanshen tablet + ISO), low-dose SMY group (SMYL; received 3.26 g/kg SMY + ISO), and high-dose SMY group (SMYH; received 13.04 g/kg SMY + ISO). SMY and compounded Danshen tablets were administered by gavage in advance for 7 days. On the 8^th^ day, all groups except the blank group were administered a subcutaneous injection of ISO for 7 days. The blank and model groups were administered the same dose of normal saline for 14 d.

#### 2.3.2. Echocardiography

On the 15^th^ day of the experiment, the rats were anaesthetized with 2% isoflurane using a small-animal inhalation gas anaesthesia machine (Midmark Group, Inc. no. VMR). Echocardiography was performed with a high-end portable colour doppler diagnostic instrument (GE Medical Systems Israel Ltd. Israel, no. VIVID I). Cardiac function was assessed by estimating the left ventricular internal diameter at end-diastole (LVIDd), left ventricular internal diameter at end-systole (LVIDs), left ventricular fractional shortening (FS%), and ejection fraction (EF%).

#### 2.3.3. Body Weight (BW), Ventricle Weight (VW), and Heart Weight Index (VW/BW) Ratio

The BW of each group of rats was measured on day 14. After each rat was sacrificed, the heart was immediately removed and placed in cold normal saline to wash off the blood. The filter paper was used to absorb the water, the VW was measured, and the VW/BW ratio was calculated.

#### 2.3.4. Masson's Staining

Cardiac tissues were fixed in 4% paraformaldehyde, embedded in paraffin after being dehydrated in a graded ethanol series, and sectioned at a thickness of 5 *μ*m. The sections were stained with Masson's trichrome for fibrosis detection. The images were visualized with an optical microscope (Olympus, Japan).

#### 2.3.5. ELISA

The animals were fasted for 12 hours after the last treatment, and 6 rats in each group were selected for further study. Rats were anaesthetized by inhalation of isoflurane using a small-animal anaesthesia machine (Shanghai Yuyan Scientific Instrument Co., Ltd.), blood was collected from the abdominal aorta, and the rats were sacrificed. The blood was placed in a centrifuge and centrifuged at 3000 rpm for 15 minutes, and serum and heart tissue were collected for subsequent experiments. FFA and cAMP ELISA kits were used to determine the contents of FFA and cAMP in rat serum and heart tissue.

#### 2.3.6. Analysis of Serum Biochemical Indexes

TG, T-CHO, LDL-C, HDL-C, ATP, and glucose kits were used to measure the contents of TG, T-CHO, LDL-C, and HDL-C in the rat serum according to the manufacturer's instructions.

#### 2.3.7. Quantitative Real-Time Polymerase Chain Reaction (qRT-PCR)

Total RNA was extracted from rat myocardial tissues by using a high-purity total RNA rapid extraction kit (BioTeke Corporation, Beijing). According to the manufacturer's (BioTeke Corporation, Beijing) instructions, total RNA was reverse transcribed into cDNA for qPCR using the BioTeke Super RT Kit. Roche FastStart Universal SYBR Green Master Mix (Rox) was used for qRT-PCR through the first-step system. The mRNA level of each gene was normalized to the mRNA level of *β*-actin. The PCR conditions were as follows: initial activation at 50°C for 2 minutes and activation at 95°C for 10 minutes, followed by denaturation at 95°C for 15 s, and annealing and extension at 60°C for 1 minute for 40 cycles. The primers used for real-time PCR are listed in Table 1.

#### 2.3.8. Sequencing of 16S rDNA Amplicons in Rat Faeces

The faeces of the rats in the blank, model, and SMYH groups were collected, with 10 rats in each group, which were collected from the last 3 days of the experiment and stored at −80°C. The CTAB or SDS method was used to extract genomic DNA from rat intestinal faeces. According to the sequencing region selected, specific primers with barcodes and high-fidelity DNA polymerase were used for PCR amplification of the selected V3-V4 variable regions. A QuantiFluor™-ST blue fluorescence quantitative system (Promega) was used to detect and quantify the PCR-amplified products. According to the sequencing volume requirements of each sample, the corresponding mixing ratio was determined. An NEB Next® Ultra™ DNA Library Prep Kit was used for library construction. The constructed library was subjected to quality inspection using an Agilent Bioanalyzer 2100 and Qubit. After the library quality inspection was performed, the library was analysed on a computer.

#### 2.3.9. Western Blotting

The heart tissue was lysed in ice-cold RIPA lysis buffer, and the protein concentration was quantified by the Bradford method (TransGen Biotech, China). The samples (30 *μ*g of protein) were then separated on a 10% SDS-PAGE gel. The separated proteins were transferred to a PVDF membrane. Then, the PVDF membrane was incubated with *β*-actin (1 : 5000), ADCY1 (1 : 500), PKA (1 : 500), Glut4 (1 : 500), Glut1 (1 : 500), CPT1A (1 : 500), PPAR*γ* (1 : 500), PPAR*α* (1 : 500), and HIF1*α* (1 : 500) antibodies overnight at 4°C. After washing with TBST, an appropriate antirabbit or antimouse IgG antibody was added to the PVDF membrane, which was incubated at room temperature for 1 hour. After washing with TBST, an enhanced chemiluminescence kit (ECL, Bioss, China) was used, and images were obtained with a gel imager (Aplegen, USA) to visualize the signal of each sample. ImageJ software was used to measure the intensity of each protein band (relative expression = target expression/*β*-actin expression).

#### 2.3.10. Statistical Analysis

Differences across groups were assessed by one-way analysis of variance. The results are expressed as the mean ± SEM. *p* values less than 0.05, 0.01, and 0.001 were considered to indicate a significant effect.

## 3. Results

### 3.1. Evaluation of the Cardiac Function by Echocardiography

**Figure 1:**
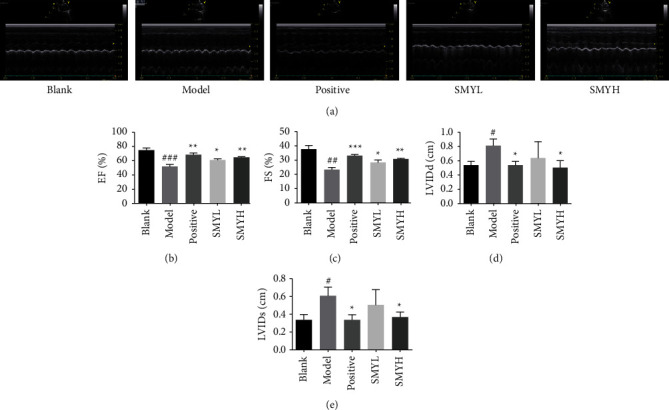
In ISO-induced myocardial hypertrophy, SMY can restore cardiac function. (a) Representative echocardiograms of rats with myocardial hypertrophy after treatment with different concentrations of SMY. (b) EF%. (c) FS%. (d) LVIDd. (e) LVIDs. *n* = 3 rats per group. The data are expressed as the mean ± SEM; *p* < 0.05 was considered significant (^#^*p* < 0.05, ^##^*p* < 0.01, and ^###^*p* < 0.001 compared with the blank group; ^*∗*^*p* < 0.05, ^∗∗^*p* < 0.01, and ^∗∗∗^*p* < 0.01 compared with the model group).

The EF%, FS%, LVIDd, and left ventricular end-systolic dimensions (LVEDs) reflect changes in the heart function. The results showed that compared with the blank group, the EF%, and FS% of the model group were decreased (*p* < 0.001, *p* < 0.01), indicating that the myocardial contractile function was significantly decreased and that the LVIDd and LVIDs were increased (*p* < 0.05). The LVIDd and LVIDs of the positive, SMYL, and SMYH groups were lower than those of the model group (*p* < 0.05), and there was no significant difference in the SMYL group; compared with the model group, the EF% and FS% of each group showed an upward trend (*p* < 0.05, *p* < 0.01) (Figure 1). These results suggest that the cardiac function in ISO-induced myocardial hypertrophy model rats is reduced and that SMY improves the cardiac function.

### 3.2. BW, VW, and the VW/BW Ratio

**Figure 2:**
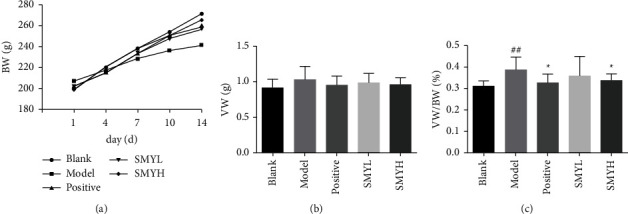
The effect of SMY on the BW, VW, and VW/BW ratio in rats with ISO-induced myocardial hypertrophy. (a) The weight of the rats at 5 experimental time points. (b) The VW of rats from each group. (c) The VW/BW ratio of rats in each group. *n* = 10 rats per group. The data are presented as the mean ± SEM; *p* < 0.05 was considered significant (^#^*p* < 0.05 and ^##^*p* < 0.01 compared with the blank group; ^*∗*^*p* < 0.05 compared with the model group).

The BW of rats in the 5 groups increased, among which the increase in the blank group was the most obvious and the increase in the model group was the slowest. Compared with the model group, the BW of the positive, SMYL, and SMYH groups increased and the weight of the SMYH group was significantly higher than that of the SMYL group. Compared with the blank group, the VW and VW/BW ratio of the model group increased (*p* < 0.01). Compared with the model group, the VW and VW/BW ratios of the positive, SMYL, and SMYH groups were all decreased (*p* < 0.05) (Figure 2). The results showed that SMY could inhibit the progression of cardiac hypertrophy.

### 3.3. Effects of SMY on Myocardial Tissue Morphology

**Figure 3:**
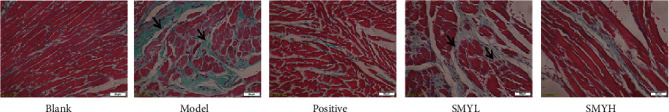
SMY inhibits ISO-induced hypertrophy-related myocardial inflammation and fibrosis. *n* = 3 rats per group; scale bar, 50 *μ*m. The black arrows indicate infiltration by inflammatory substances, cell necrosis, blue collagen, and partial fibrosis.

In the blank group, the structure of myocardial cells was regular, the fibres were tightly arranged, and there was no cell necrosis. Myocardial cells in the model group showed swelling, necrosis (arrow), and inflammatory infiltration, and a large number of blue flaky collagen fibres were seen (arrow). Myocardial tissue fibrosis was severe and fibre arrangement was disordered. The degree of damage and alterations in fibre structure and the morphology of myocardial cells were significantly alleviated in the positive, SMYL, and SMYH groups compared with the model group (Figure 3). The results show that SMYH can ameliorate ISO-induced myocardial hypertrophy, injury, and fibrosis in rats.

### 3.4. The Contents of FFAs and cAMP in the Heart Tissue and Serum of Rats in Each Group

**Figure 4:**
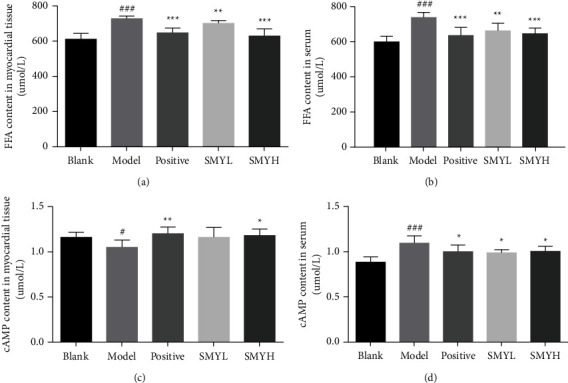
Effect of SMY on the contents of FFAs and cAMP in the myocardial tissue and serum of rats with ISO-induced myocardial hypertrophy. (a) FFA content in myocardial tissues. (b) FFA content in the serum. (c) cAMP content in myocardial tissues. (d) cAMP content in the serum. *n* = 6 rats per group. The data are presented as the mean ± SEM; *p* < 0.05 was considered significant (^#^*p* < 0.05 and ^###^*p* < 0.001 compared with the blank group; ^*∗*^*p* < 0.05, ^∗∗^*p* < 0.01 and ^∗∗∗^*p* < 0.01 compared with the model group).

Compared with the blank group, the levels of FFA in myocardial tissues and serum in the model group were significantly increased (*p* < 0.001, *p* < 0.001), the content of cAMP in myocardial tissue was decreased (*p* < 0.05), and the content of cAMP in serum was significantly increased (*p* < 0.05, *p* < 0.001). Compared with the model group, the levels of FFA in the myocardial tissue and serum in the positive group were significantly decreased (*p* < 0.001, *p* < 0.001), the content of cAMP in the myocardial tissue was increased (*p* < 0.01), and the content of cAMP in the serum was decreased (*p* < 0.05). After SMY treatment, the levels of FFA in the myocardial tissue and serum of rats in the SMYL group were significantly improved (*p* < 0.01, *p* < 0.01), the content of cAMP in myocardial tissues was increased and the content of cAMP in serum was decreased (*p* < 0.05). FFA levels in the myocardial tissue and serum of rats in the SMYH group were significantly improved (*p* < 0.001, *p* < 0.001), cAMP content in myocardial tissue increased (*p* < 0.05), and cAMP content in serum decreased (*p* < 0.05) (Figure 4). The results showed that SMYH could regulate lipid and energy metabolism and improve cardiac function.

### 3.5. Effect of SMY on TG, T-CHO, LDL-C, HDL-C, ATP, and Glucose Contents in the Serum of Rats with Cardiac Hypertrophy

**Figure 5:**
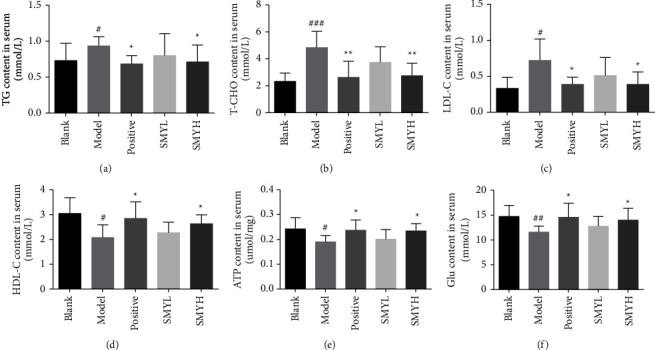
The effect of SMY on the TG, T-CHO, LDL-C, HDL-C, ATP, and glucose contents in the serum of rats with ISO-induced myocardial hypertrophy. (a) TG content in the serum. *n* = 8 rats per group. (b) T-CHO content in the serum. *n* = 8 rats per group. (c) LDL-C content in the serum. *n* = 6 rats per group. (d) Serum HDL-C content. *n* = 6 rats per group. (e) ATP content in the serum. *n* = 6 rats per group. (f) Serum glucose content. *n* = 6 rats per group. The data are presented as the mean ± SEM; *p* < 0.05 was considered significant (^#^*p* < 0.05 and ^###^*p* < 0.001 compared with the blank group; ^*∗*^*p* < 0.05 and ^∗∗^*p* < 0.01 compared with the model group).

Compared with the blank group, the levels of TG, T-CHO, and LDL-C in the model group were significantly increased (*p* < 0.05, *p* < 0.001, *p* < 0.05), while the levels of HDL-C, ATP, and Glu in the model group were significantly decreased (*p* < 0.05, *p* < 0.05, *p* < 0.01). Compared with the model group, the contents of TG, T-CHO, and LDL-C in the positive group were significantly decreased (*p* < 0.05, *p* < 0.01, *p* < 0.05), while the contents of HDL-C, ATP, and Glu were increased (*p* < 0.05, *p* < 0.05, *p* < 0.05). The contents of TG, T-CHO, and LDL-C in the SMYH group were significantly decreased (*p* < 0.05, *p* < 0.01, *p* < 0.05), and the contents of HDL-C, ATP, and Glu were increased (*p* < 0.05, *p* < 0.05, *p* < 0.05). Although there was a change in the stool in the SMYL group, there was no significant difference (Figure 5). This result shows that SMYH can improve blood lipid metabolism, regulate energy metabolism, and increase the cardiac energy supply.

### 3.6. Effect of SMY on the Expression of the ANP, BNP, and *β*-MHC Genes in Rats with Myocardial Hypertrophy

**Figure 6:**
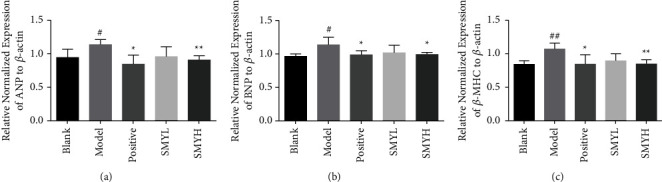
The effect of SMY on the relative gene expression of ANP, BNP, and *β*-MHC in the myocardial tissue of rats with ISO-induced myocardial hypertrophy. (a) ANP expression. (b) BNP expression. (c) *β*-MHC expression. *n* = 4 rats per group. The data are presented as the mean ± SEM; *p* < 0.05 was considered significant (^#^*p* < 0.05 and ^##^*p* < 0.01 compared with the blank group; ^*∗*^*p* < 0.05 and ^∗∗^*p* < 0.01 compared with the model group).

Compared with the blank group, the relative gene expression levels of ANP, BNP, and *β*-MHC in the myocardial tissue of the model group were significantly increased (*p* < 0.05, *p* < 0.05, *p* < 0.01). Compared with the model group, the expression levels of ANP, BNP, and *β*-MHC in the SMYL group decreased, but the difference was not significant, and the expressions in the positive group and the SMYH group decreased significantly (*p* < 0.05, *p* < 0.01) (Figure 6). The results further showed that SMYH significantly attenuated ISO-induced myocardial injury and hypertrophy in rats.

### 3.7. Effect of SMY on 16S rDNA Amplicon Sequencing in the Faeces of Rats with Cardiac Hypertrophy

#### 3.7.1. Venn Diagram of OTU Distribution

**Figure 7:**
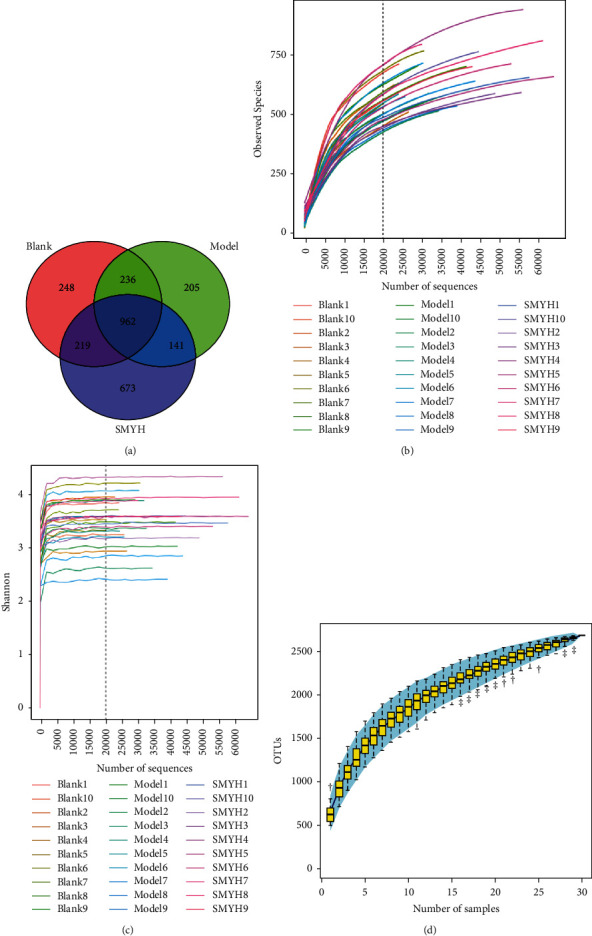
Venn diagram of OTU distribution and curves. (a) Venn diagram. The different colours represent the different groups. Red indicates the blank group, green indicates the model group, and blue indicates the SMYH group. (b) Rarefaction curve. The *x* axis represents the amount of randomly selected sequencing data and the *y* axis represents the number of OTUs observed. (c) Shannon curve. The *x* axis represents the amount of randomly selected sequencing data and the *y* axis represents the Shannon index. (d) Species accumulation curves. The *x* axis represents the sample size and the *y* axis represents the number of OTUs after sampling. *n* = 10 rats per group.

To explore the degree of difference in OTU types among three samples from the blank, model, and SMYH groups, the unique OTUs in each group and the OTUs shared with other groups were identified and a Venn diagram was generated. The degree of overlap between the circles of different colours was used to intuitively reflect the similarity between OTUs. The OTUs in the overlapping part are shared OTUs, and those in the nonoverlapping part are unique OTUs to each group. The total number of OTUs among the three groups was 962 (Figure 7(a)).

#### 3.7.2. Rarefaction, Shannon, and Species Accumulation Curves

A Shannon curve was constructed based on the microbial diversity index of each sample at different sequencing depths. Species accumulation curves were used to describe the increase in species diversity as the sample size increased. In biodiversity and community surveys, these curves are widely used to assess whether the sample size is sufficient and to estimate species richness. A rarefaction curve, Shannon curve, and species accumulation curve were constructed for sequencing data from the blank, model, and SMYH group samples, and the results showed that the species richness was good and that subsequent experiments could be performed (Figures 7(b)–7(d)).

#### 3.7.3. *α* Diversity Analysis

**Figure 8:**
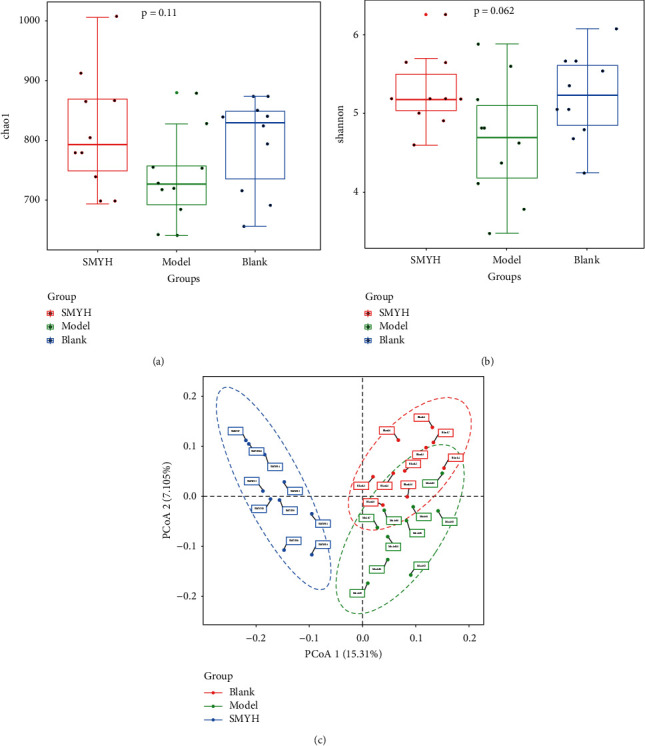
Analysis of differences in the *α* diversity index and *β* diversity, as determined by PCoA, between groups. (a) Chao1 index. (b) Shannon index. (a, b) The first quartile (Q1) in the figure, also known as the “lower quartile”, is the 25th percentile of all the values in the sample in descending order. The second quartile (Q2), also known as the “median”, is the 50th percentile of all values in the sample in descending order. The third quartile (Q3), also known as the “upper quartile”, is the 75^th^ percentile of all values in the sample in descending order. (c) PCoA of *β* diversity. The *x* axis represents the first principal component, the *y* axis represents the second principal component, and the percentage represents the contribution to the difference between samples. *n* = 10 rats per group.

When studying changes in the structure of microbial communities, *α* diversity is usually used to reflect the abundance and diversity of microbial communities. Commonly used indexes are the Chao1 and Shannon indexes. The Chao1 index is commonly used to evaluate the number of species in a community, i.e., the community richness. The larger the Chao1 index is, the greater the abundance of the community. The Shannon index reflects the diversity of microorganisms and is often used to analyse the structure of microbial communities. As a community diversity index, the smaller the Shannon index is, the higher the diversity of the community. Compared with those of the blank group, the Chao1, and Shannon indexes of the model group were decreased, indicating that ISO treatment disrupted the richness and diversity of the microbial community in the rat colon. These indexes were increased in the SMYH group (Figures 8(a), 8(b)).

#### 3.7.4. *β* Diversity Analysis

Principal coordinate analysis (PCoA) was used to assess the similarities and differences in community composition between samples. When the distance between the samples is smaller, the species composition structure is more similar. The results for PCoA1 showed a difference of 12.7% between the blank, model, and SMYH groups, and the results for PCoA2 showed a difference of 7.105%, indicating good separation of the three groups (Figure 8(c)).

#### 3.7.5. Analysis of the Abundance of Different Species between Groups

**Figure 9:**
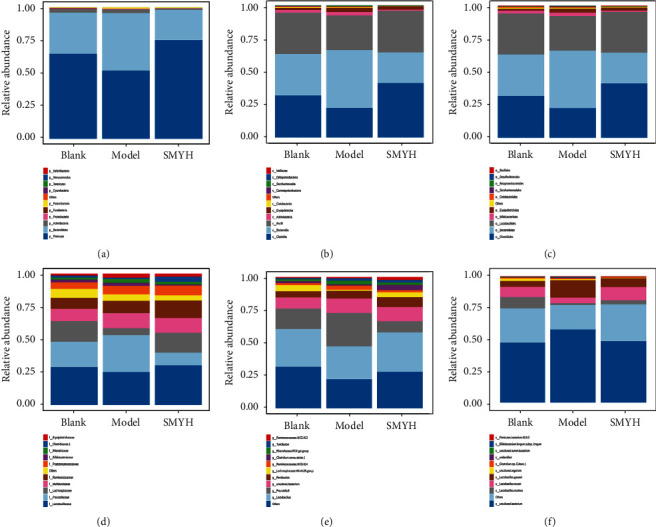
Relative abundance of species and biomarkers in samples from each group. (a) Relative abundance of bacteria in each group at the phylum level. (b) Relative abundance of bacteria in each group at the class level. (c) Relative abundance of bacteria in each group at the order level. (d) Relative abundance of bacteria in each group at the family level. (e) Relative abundance of bacteria in each group at the genus level. (f) Relative abundance of bacteria in each group at the species level. (a-f) The different colours represent different species, as indicated in the legend on the right, the horizontal axis represents the sample or group, and the vertical axis represents the relative abundance of different species.

By comparing the abundance of bacteria between the blank, model, and SMYH groups, it was found that the overall intestinal flora structure was similar among the three groups of rats. At the phylum level, *p_Firmicutes*, *p_Bacteroidetes,* and *p_Actinobacteria* were the main flora. The results showed that compared with that in the model group, the abundance of *p_Bacteroidetes* and *p_Actinobacteria* in the SHYH group was significantly decreased and the abundance of *p_Firmicutes* in the SHYH group was increased. *p_Firmicutes* and *p_Bacteroides* are important indicators of nutrient absorption and energy metabolism, and a high proportion of *p_Firmicutes* can help the body absorb more nutrients from food (Figure 9(a)). At the class level, the relative abundance of *c_Bacilli* was decreased and the relative abundance of *c_Erysipelotrichia* and *c_Saccharimonadia* was increased in the model group, while the SMYH group showed the opposite trend (Figure 9(b)). At the order level, the relative abundance of *o_Lactobacillales* was decreased, and the relative abundance of *o_Erysipelotrichales* and *o_Saccharimonadales* was increased in the model group. The SMYH group exhibited the opposite trend (Figure 9(c)). At the family level, the relative abundance of *f_Lactobacillaceae* and *f_Lachnospiraceae* was decreased, and the relative abundance of *f_Erysipelotrichaceae*, *f_Muribaculaceae,* and *f_Rikenellaceae* was increased in the model group, while the SMYH group showed the opposite trend (Figure 9(d)). At the genus level, the relative abundance of the *g_Lactobacillus* and *g_Lachnospiraceae NK4A136 groups* was decreased and the relative abundance of the *g_uncultured bacterium*, *g_Ruminococcaceae UCG-014,* and *g_Rikenellaceae RC9* gut groups was increased in the model group, while the SMYH group showed the opposite trend (Figure 9(e)). At the species level, the relative abundance of *s_Lactobacillus murinus* and *s_uncultured rumen bacteria* was decreased and the relative abundance of *s_uncultured bacterium* and *s_Lactobacillus gasseri* was increased in the model group, while the SMYH group showed the opposite trend (Figure 9(f)).

#### 3.7.6. Function Prediction

**Figure 10:**
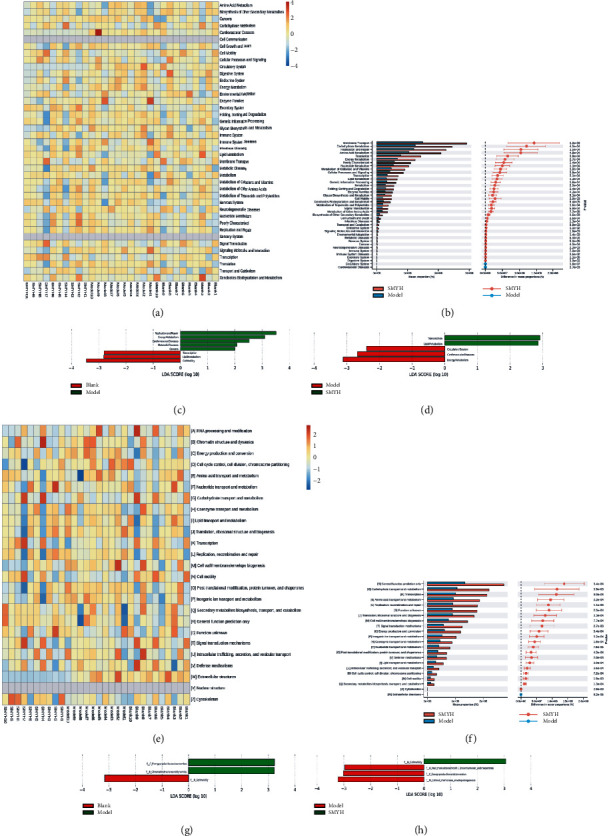
Function prediction by KEGG and COG analyses. (a) Heatmap of KEGG terms. The different colours represent the different functional items, as indicated in the legend on the right; the horizontal axis represents the sample or group, and the vertical axis represents the relative abundance of bacteria associated with each functional term. (b) STAMP plot of the KEGG results. The left figure shows the abundance ratio of bacteria associated with different functional terms between two samples or two groups. The middle figure indicates the difference ratio within a 95% confidence interval. The rightmost value is the *p* value, and a *p* value <0.05 indicates a significant difference. (c) LDA score obtained by LefSe analysis of the KEGG results. Red represents the blank group and the green represents the model group. (d) LDA score obtained by LefSe analysis of the KEGG results. Red represents the model group, and the green represents the SMYH group. (e) Heatmap of COG terms. (f) STAMP plot of the COG results. (g) LDA score obtained by LefSe analysis of the COG results. (h) LDA score obtained by LefSe analysis of the COG results. *n* = 10 rats per group.

KEGG analysis is an effective method for studying the adaptations in the metabolic function of a community to environmental changes. COG, a prokaryotic homologous protein cluster database, is a commonly used protein function classification database for prokaryotes. It is complementary to KEGG and reveals the function of bacteria more comprehensively. The functional composition of each group was analysed. As shown in the heatmap of KEGG and COG terms, the main terms were amino acid metabolism, nucleotide metabolism, energy metabolism, carbohydrate metabolism, cofactor and vitamin metabolism, and lipid metabolism (Figures 10(a), 10(b), 10(e), and 10(f)). Based on LefSe analysis of KEGG and COG terms, we ultimately identified energy metabolism, carbohydrate metabolism, and lipid metabolism (Figures 10(c), 10(d), 10(g), and 10(h)).

### 3.8. Effect of SMY on the Expression of HIF1*α*/PPAR Signalling Pathway-Related Proteins in Rats with Cardiac Hypertrophy

**Figure 11:**
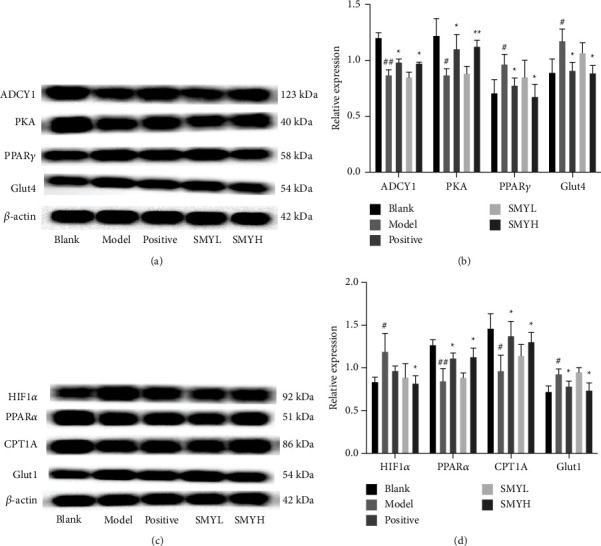
SMY regulates the key signalling pathway in rats with ISO-induced myocardial hypertrophy. (a) ADCY1, PKA, PPAR*γ,* and Glut4 levels. (b) Relative expression of ADCY1, PKA, PPAR*γ,* and Glut4. (c) HIF1ɑ, PPARɑ, CPT1A, and Glut1 levels. (d) Relative expression of HIF1*α*, PPAR*α*, CPT1A, and Glut1. *n* = 3 rats per group. The data are presented as the mean ± SEM; *p* < 0.05 is considered significant (^#^*p* < 0.05, ^##^*p* < 0.01 and ^###^*p* < 0.001 compared with the blank group; ^*∗*^*p* < 0.05, ^∗∗^*p* < 0.01 and ^∗∗∗^*p* < 0.001 compared with the model group).

Based on KEGG functional enrichment and COG analysis, we initially identified energy metabolism-related pathways. To further analyse the therapeutic effect of SMY on myocardial hypertrophy in rats, we assessed the expression of HIF1*α*/PPAR signalling pathway-related proteins. We found that in the model group, the protein expression levels of ADCY1, PKA, PPAR*α*, and CPT1A in myocardial tissue were significantly decreased (*p* < 0.01, *p* < 0.05, *p* < 0.01, *p* < 0.05) (Figures 11(a)–11(d)), as well as that the protein expression levels of HIF1*α*, PPAR*γ*, Glut4, and Glut1 were increased (*p* < 0.05), the positive group and the SMYH group showed the opposite trend (*p* < 0.05). Although there was a change in the SMYL group, it was not statistically significant (Figures 11(a)–11(d)). The results show that SMYH can inhibit the protein expression of HIF1*α*/PPAR signalling pathway-related proteins and activate the PKA signalling pathway, thereby alleviating energy metabolism disorders in rats with myocardial hypertrophy. The results show that SMYH can inhibit the protein expression of HIF1*α*/PPAR signalling pathway-related proteins and activate the PKA signalling pathway, thereby alleviating energy metabolism disorders in rats with myocardial hypertrophy.

## 4. Discussion

**Figure 12:**
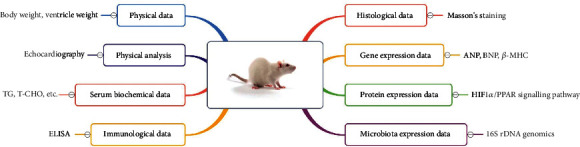
Study design of the protective effect and mechanism of SMY on ISO-induced myocardial hypertrophy in rats.

We conducted relevant experiments according to the research design (Figure 12). In our study, an in vivo model of myocardial hypertrophy was established in rats by subcutaneous injection of the *β*-adrenoceptor agonist ISO. Similar to earlier reports, the expression levels of the myocardial hypertrophy markers ANP, BNP, and *β*-MHC, and the VW/BW ratio were all increased after ISO treatment [17]. Several studies have reported that SMY has a protective effect on cardiovascular disease, and the mechanism of action includes antiapoptosis, improvement of cardiac systolic and diastolic function, and protection of the mitochondrial structure. It also has a positive effect on myocardial hypertrophy [18, 19]. Our study revealed that SMY treatment reduced VW/BW, blood lipids, and myocardial hypertrophy marker levels and improved myocardial histopathological damage, which was consistent with the results of previous studies [20, 21]. In conclusion, SMY protects against ISO-induced myocardial hypertrophy.

Some studies have suggested that the gut has a certain influence on the pathogenesis of myocardial hypertrophy. The gut-cardiac axis theory suggests that reduced cardiac output and systemic congestion can lead to intestinal ischaemia and oedema, leading to increased bacterial translocation and increased endotoxins, which can lead to intestinal ischaemia and oedema and aggravate the underlying inflammatory response in patients with myocardial hypertrophy. Persistent myocardial hypertrophy induces cardiac remodelling, heart failure, and ultimately death [22]. Therefore, there is still great potential to study the role of the gut-cardiac axis in myocardial hypertrophy.

In this study, we used 16S rDNA sequencing technology to observe the changes in the composition and structure of intestinal flora during the occurrence and development of myocardial hypertrophy. The results of this study showed that the diversity and richness of gut microbiota were destroyed during the development of ISO-induced myocardial hypertrophy, which was basically consistent with previous studies [23, 24], suggesting that intestinal microbiota may occur in patients with myocardial hypertrophy. According to the results of the PCoA analysis in this study, myocardial hypertrophy was accompanied by changes in the overall structure of the gut microbiota.

In this study, it was found that the occurrence of myocardial hypertrophy may be related to the increase in the abundance of *Prevotella 9* and *Ruminococcaceae* and the decrease in the abundance of *Clostridium* and *Lactobacillus*; after administration of SMY, the abundance of the above bacteria was reversed, indicating that SMY intervention can induce different degrees of improvement in the diversity and abundance of intestinal flora, significantly reduce pathogenic bacteria, and increase the abundance of beneficial bacteria. Patients with myocardial hypertrophy have a reduced amount of the gut microbiota metabolite butyrate, which can break down dietary fibres into short-chain fatty acids (SCFAs) through glycation. *Clostridium* is the bacterium that metabolizes butyrate the most [25]. In this study, SMY positively regulated the abundance of *Clostridium*, thereby increasing the level of SCFAs. SCFAs are byproducts of dietary fibre metabolism by bacteria in the distal small intestine and caecum, have anti-inflammatory effects, and promote the growth of beneficial gut bacteria [26], and the increased content of SCFAs may be related to the improvement of intestinal barrier function [27]. This study revealed that SMY can treat myocardial hypertrophy by improving intestinal barrier function. *Prevotella 9* has been reported to have a certain effect on cardiovascular disease, and the phosphoadenosine sulfate reductase and superoxide reductase encoded by *Prevotella* may help trigger and maintain intestinal inflammation [28]. Studies have also linked increased levels of *Prevotella* to inflammatory diseases such as metabolic syndrome [29]. *Prevotella* can induce inflammation by producing succinate and elevated levels of succinate have been found in experimental models of metabolic and inflammatory diseases, which induce the development of heart failure [30]. Hypertrophy may also contribute to the development of heart failure by enhancing the inflammatory response. Other studies define *Prevotella* as beneficial bacteria that drive the biosynthesis of branched-chain amino acids (BCAAs) in the gut microbiota [31,32]. However, Du et al. found that Baoyuan decoction positively regulated the abundance of the intestinal flora *Prevotella 9* in rats with heart failure, which was inconsistent with the results of this study [33].

*Lactobacillus* is considered to be an essential bacteria in a healthy state, and it is an important probiotic necessary to protect the intestinal barrier, improve immune function, and inhibit the growth of pathogenic bacteria [34]. Studies have demonstrated that *Lactobacillus gasseri*, a probiotic, can reduce blood lipids and bile acids in hypercholesterolaemia rats [35]. Another study revealed that oral administration of *Lactobacillus rhamnosus* in mice significantly reduced triglyceride and serum cholesterol levels. *Lactic acid bacteria* can inhibit the development of myocardial hypertrophy and heart failure in rats by reducing plasma adiponectin levels and improving ventricular systolic and diastolic function [36]. In the present study, *Lactobacilli* increased in the gut of rats after SMY administration and we found that maintaining healthy levels of *Lactobacilli* indirectly affected blood lipid levels.

In addition, along with the change in the intestinal flora, its gene function must change accordingly. In this study, the gene function difference analysis of gut microbiota showed that the amino acid metabolism function of the model group was relatively reduced, which indicated that the energy generation ability was impaired, and the biosynthesis of nucleotides was also reduced. Since *Lactobacillus* can promote the metabolism of essential human molecules such as amino acids, proteins, and nucleotides [34]; the downregulation of these metabolic functions in this study may be related to the downregulation of *Lactobacillus* abundance, indicating that the decrease in *Lactobacillus* abundance may be affected by body metabolism, thereby aggravating myocardial hypertrophy. Furthermore, based on KEGG functional prediction analysis, we predicted that the antimyocardial hypertrophic effect of SMY may be related to the regulation of abnormal energy metabolism. The intestinal flora plays an important role in controlling the host's energy metabolism. The intestinal flora fermentatively degrades polysaccharides in the body to provide energy for the host [37]. Polysaccharides can be digested and decomposed into SCFAs by intestinal flora, such as *Clostridium butyricum*. Under normal physiological conditions, the major substrate of myocardial energy metabolism is fatty acids, and during the development of myocardial hypertrophy, myocardial energy utilization may be gradually switched from fatty acids to glucose to reduce oxygen consumption and maintain the same level of ATP production [38]. Studies have shown that SMY protects against myocardial ischaemia-reperfusion injury and doxorubicin-induced cardiomyopathy and is related to improving myocardial energy metabolism [39]. However, patients with myocardial hypertrophy have very high myocardial oxygen consumption and exhibit energy deficits (30 to 40% reduction in ATP content compared with healthy hearts) due to altered availability of energy substrates and impaired mitochondrial oxidative metabolism [40].

Changes in glucose and lipid metabolism may be caused by the activation of HIF1*α*, which is a key regulator of glucose and lipid metabolism [41], and when myocardial hypertrophy occurs, myocardial hypoxia activates HIF1*α*. Activated HIF1*α* binds to the Glut1 enhancer, resulting in the abundant expression of Glut1, which increases glucose uptake. After HIF1*α* is activated, it is involved in multiple physiological processes, including glucose uptake, glycolysis, angiogenesis, and cell survival. HIF1*α* plays a key role in the enhancement of cardiac energy metabolism [42], and its activation may switch from fatty acid metabolism to glucose metabolism through a PPAR*α*-mediated pathway [43]. Studies have revealed that chrysanthemum extract can inhibit the expression of HIF1*α* in the myocardium and increase the expression of myocardial PPAR*α*, as well as that myocardial energy metabolism may be mainly regulated by the HIF1*α*-PPAR pathway [44]. This result is consistent with that found in the present study. In this study, HIF1*α* expression was upregulated and PPARa expression was downregulated in the model group and this phenomenon was reversed by SMY treatment.

Studies have shown that Shengmai injection (SMI) can inhibit the expression of Glut4 in cells and by activating the expression of PPAR*α* and CPT1A, it can promote fatty acid oxidation in mitochondria and improve myocardial energy metabolism [45]. PPAR*α* can regulate the expression of downstream CPT1A [46], and downregulation of CPT1A expression can reduce fatty acid *β* oxidation in the myocardium [47]. The expression of CPT1A in the model group in this study decreased, indicating that the utilization of fatty acids is hindered, resulting in an increase in FFA. In addition to fatty acid oxidation, glycolysis is an alternative source of ATP production [48], glucose is taken up by cardiomyocytes via Glut1 and Glut4 [49], and Glut1 and Glut4 protein expression was upregulated in the model group in this study, indicating that transport from blood to myocardial tissues increases glucose. More importantly, SMY treatment alleviated the disorder of glycolipid metabolism by upregulating CPT1A and downregulating Glut1 and Glut4, resulting in decreased FFA and increased Glu. Therefore, we concluded that SMY may ameliorate ISO-induced myocardial hypertrophy by regulating the HIF1a-PPAR pathway and the expression of downstream glycolipid metabolism-related proteins, thereby alleviating the disturbance of energy metabolism.

## 5. Conclusions

Before this study, we conducted a preliminary experiment, mainly to screen the dose of SMY in rats. The dose of SMYH used in this study had no toxic or side effects in rats. In summary, the results of this study showed that SMY reduced myocardial fibrosis in rats, increased EF% and FS%, and protected rat myocardium from damage caused by ISO-induced myocardial hypertrophy. In addition, various biomarkers related to hypertrophy, such as the levels of ANP, BNP, and *β*-MHC, indicated that SMY treatment improved heart function at the genetic level. Moreover, SMY was shown to regulate changes in the intestinal microflora of rats and improve resistance to myocardial hypertrophy. Western blot analysis showed that SMY inhibited the protein expression of HIF1*α*/PPAR signalling pathway-related proteins to activate the PKA signalling pathway, thereby ameliorating energy metabolism disorder in rats with myocardial hypertrophy. Therefore, SMY may prevent or inhibit myocardial hypertrophy.

## Figures and Tables

**Table 1:** Primer sequences used for real-time PCR.

Gene	Forward primer	Reverse primer
ANP	TCG AGC AGA TCG CAA AAG ATC	CAC ACT AAA CCA CTC ATC TAC
BNP	AAG CTG CTG GAG CTG ATA AGA	GTT ACA GCC CAA ACG ACT GAC
*β*-MHC	CTG GCA CCG TGG ACT ACA AC	CGC ACA AAG TGA GGA TAG GGT
*β*-actin	CAA GCT TAA GGT TAA TCA GG	ACA TTA GGC CAT CGA ATC CG

## Data Availability

All data for the results of this study can be obtained from the author, Mo Kan, at 512904695@qq.com.
